# PReS-FINAL-2141: Clinical features, therapeutic interventions and outcome of 362 patients with macrophage activation syndrome enrolled in a multinational survey

**DOI:** 10.1186/1546-0096-11-S2-P153

**Published:** 2013-12-05

**Authors:** F Minoia, S Davì, A Horne, A Consolaro, S Rosina, Z Davidsone, C De Cunto, J De Inocencio, E Eisenstein, G Espada, M Fishbach, M Frosch, R Gallizzi, ML Gamir, T Griffin, A Grom, T Hennon, G Horneff, Z Huasong, N Ruperto, A Martini, RQ Cron, A Ravelli

**Affiliations:** 1 Istituto Giannina Gaslini, Genoa, Italy; 2 Childhood Cancer Research Unit, Dep. Of Women's and Children's Health, Karolinska Institutet, Stockholm, Sweden; 3 International Investigator Consortium for MAS Diagnostic Criteria (IICMASDC), Riga, Latvia; 4 IICMASDC, Buenos Aires, Argentina; 5 IICMASDC, Madrid, Spain; 6 IICMASDC, Jerusalem, Israel; 7 IICMASDC, Strasbourg, France; 8 IICMASDC, Muenster, Germany; 9 IICMASDC, Messina, Italy; 10 IICMASDC, Cincinnati, USA; 11 IICMASDC, Buffalo, USA; 12 IICMASDC, Sankt Augustin, Germany; 13 IICMASDC, Guangzhou, China; 14 University of Alabama at Birmingham, Birmingham, USA

## Introduction

A multinational collaborative effort aimed to develop a new set of criteria for macrophage activation syndrome (MAS) complicating systemic juvenile idiopathic arthritis (sjia) is ongoing. The data-collection phase of the project has been recently completed

## Objectives

To describe the demographic, clinical, laboratory, and histopathologic features, therapeutic interventionsand outcome of 362 children with MAS collected in the study

## Methods

Patient data were collected retrospectively in a web-based database, developed and handled at the coordinating centre (Istituto G. Gaslini, Genoa, Italy)

## Results

362 patients with sjia-associated MAS were entered in the study website by 95 investigators (78.2% paediatric rheumatologists, 21.8% paediatric hemato-oncologists) from 33 countries. 208 patients (57.5%) were females. Median age at onset of sjia was 5.3 years (IQR 2.7-10.1 years) and median disease duration at onset of MAS was 3.5 months (IQR 0.1-2.6 years); MAS occurred at onset of sjia in 77 patients (22.2%). The most frequently observed clinical features were fever (96%), liver enlargement (70%) and spleen enlargement (58%); CNS involvement was reported in 122 patients (35%) and haemorrhagic manifestations in 71 patients (20%). The main laboratory abnormalities were: hyperferritinemia, increased D-dimer and liver enzymes, falling platelet count, hypertriglyceridemia and increased LDH. The most frequently reported trigger of MAS was sjia flare (53.8%), followed by infections (37.8%) and medication toxicity (4.3%). Hyperferritinemia, increased liver enzymes, LDH, triglycerides and D-dimer and falling platelet count were the sole laboratory parameters that showed a percentage change greater than 50% between pre-MAS visit and onset of MAS. Hemophagocytosis was seen in 2/3 of patients who underwent bone marrow aspirate. Therapeutic interventions included corticosteroids (97.7%), cyclosporine (61.2%), Iv Ig (36.3%), biologic medications (15.2%), etoposide (11.8%), other immunosuppressants (7.1%) and plasma exchange (4.1%). ICU admission was required in 34.9% of patients; the mortality rate was 8.1%

## Conclusion

Fever and hepatosplenomegaly were the most frequently reported clinical features. Hyperferritinemia, increased liver enzymes, LDH, triglycerides and D-dimer and falling platelet count were the most frequently observed laboratory abnormalities. These laboratory parameters also showed the greatest change between pre-MAS visit and onset of MAS. Bone marrow aspirate exhibited hemophagocytosis in 2/3 of instances. Corticosteroids and cyclosporine were the most frequently used medications.

## Disclosure of interest

None declared.

